# Global response of *Plasmodium falciparum *to hyperoxia: a combined transcriptomic and proteomic approach

**DOI:** 10.1186/1475-2875-10-4

**Published:** 2011-01-11

**Authors:** Marylin Torrentino-Madamet, Lionel Alméras, Jérôme Desplans, Yannick Le Priol, Maya Belghazi, Matthieu Pophillat, Patrick Fourquet, Yves Jammes, Daniel Parzy

**Affiliations:** 1 UMR-MD3 (Université de la Méditerranée), Antenne IRBA de Marseille (IMTSSA, Le Pharo), Allée du Médecin Colonel Eugène Jamot, BP 60109, 13262 Marseille cedex 07, France; 2 Unité de Recherche en Biologie et Epidémiologie Parasitaires (URBEP), Antenne IRBA de Marseille (IMTSSA, Le Pharo), Allée du Médecin Colonel Eugène Jamot, BP 60109, 13262 Marseille cedex 07, France; 3 Centre d'Analyse Protéomique de Marseille (CAPM), Institut Fédératif de Recherche Jean Roche, Faculté de Médecine Nord, Bd. Pierre Dramard, 13916 Marseille cedex 20, France; 4 Centre d'Immunologie de Marseille Luminy (CIML), Institut National de la Santé et de la Recherche Médicale, Centre National de la Recherche Scientifique, Université de la Méditerranée, Parc Scientifique de Luminy, 13288 Marseille Cedex 09, Marseille, France; 5 UMR-MD2, Physiologie et Physiopathologie en Conditions d'Oxygénations Extrêmes, Institut Fédératif de Recherche Jean Roche, Faculté de Médecine Nord, Bd. Pierre Dramard, 13916 Marseille cedex 20, France

## Abstract

**Background:**

Over its life cycle, the *Plasmodium falciparum *parasite is exposed to different environmental conditions, particularly to variations in O_2 _pressure. For example, the parasite circulates in human venous blood at 5% O_2 _pressure and in arterial blood, particularly in the lungs, at 13% O_2 _pressure. Moreover, the parasite is exposed to 21% O_2 _levels in the salivary glands of mosquitoes.

**Methods:**

To study the metabolic adaptation of *P. falciparum *to different oxygen pressures during the intraerythrocytic cycle, a combined approach using transcriptomic and proteomic techniques was undertaken.

**Results:**

Even though hyperoxia lengthens the parasitic cycle, significant transcriptional changes were detected in hyperoxic conditions in the late-ring stage. Using PS 6.0™ software (Ariadne Genomics) for microarray analysis, this study demonstrate up-expression of genes involved in antioxidant systems and down-expression of genes involved in the digestive vacuole metabolism and the glycolysis in favour of mitochondrial respiration. Proteomic analysis revealed increased levels of heat shock proteins, and decreased levels of glycolytic enzymes. Some of this regulation reflected post-transcriptional modifications during the hyperoxia response.

**Conclusions:**

These results seem to indicate that hyperoxia activates antioxidant defence systems in parasites to preserve the integrity of its cellular structures. Moreover, environmental constraints seem to induce an energetic metabolism adaptation of *P. falciparum*. This study provides a better understanding of the adaptive capabilities of *P. falciparum *to environmental changes and may lead to the development of novel therapeutic targets.

## Background

*Plasmodium falciparum *is a protozoan parasite responsible for the most severe form of human malaria. This infection causes between 708,000 and 1,003,000 human deaths each year, most of them occurring in African children under the age of five years [1]. Several anti-malarial agents are used for malaria treatment and prophylaxis in endemic regions. However, the expansion of drug-resistance remains a serious problem. To develop new anti-malarial drugs, a better understanding of *P. falciparum *biology is required [2]. Some unique properties of the *P. falciparum *mitochondrion indicate that its respiratory metabolism could be exploited to generate chemotherapeutic targets [3]. Indeed, atovaquone, a mitochondrial cytochrome *bc1 *complex inhibitor, is currently used in combination with proguanil (Malarone™, GlaxoSmithKline) for malaria treatment and prophylaxis [4,5].

*In vitro*, *P. falciparum *growth is maximal in limited oxygen content (0.5%-5.0% O_2_), so the parasite is considered as a microaerophilic organism. *In vivo*, the parasite life cycle involves two hosts: the mosquito and the human, during which the parasite undergoes different cellular morphological changes and experiences oxygen pressure variations. The passage from one host to another implies metabolic adaptation and changes in the ultrastructural and physiological organization of mitochondria [6-8]. In humans, the parasite is exposed to varying oxygen pressures, which can reach up to 13% O_2 _in lung capillaries [9]. In mosquitoes, the parasite is exposed to 21% O_2 _levels in salivary glands. To adapt to these environmental constraints, the parasite has developed metabolic adaptations essential for it survival. During the intraerythrocytic cycle, two metabolic pathways are the major sources of ROS (superoxide anions, hydrogen peroxide and hydroxyl radicals) on *P. falciparum *- the mitochondrial respiratory chain and haemoglobin digestion [10]. In the presence of oxygen, *P. falciparum *can produce ATP by aerobic respiration and through glycolysis. Accordingly, microaerophilic metabolism may be a metabolic adaptation to prevent oxidative stress generation [10,11]. The parasite also consumes haemoglobin in its digestive vacuole for protein biosynthesis. This metabolic pathway is a source of superoxide anions and ferriprotoporphyrin IX (FIX) accumulation [12], and thus ROS produced in the mitochondria could interact with these products of haemoglobin digestion and increase oxidative damage to the parasite cells. Therefore, *P. falciparum *has developed a preventive defence system to reduce cellular damage.

To identify metabolic pathways involved in the hyperoxia response, the effect of oxygen on *P. falciparum *was studied using high-throughput transcriptomic and proteomic analyses in the late-ring stage. These approaches were designed to minimize non-specific responses [13], and they revealed that a stress response occurs following parasite exposure to hyperoxia and that *P. falciparum *modifies the metabolism of two organelles (the mitochondrion and the digestive vacuole) as a metabolic adaptation to this environmental challenge.

## Methods

### *Plasmodium falciparum in vitro *culture

The 3D7 *P. falciparum *strain (the reference strain used for the genome sequencing project) was obtained from the Malaria Research and Reference Reagent source centre (MR4, managed by the American type culture collection). Parasitized human red blood cells (RBC type A+) were maintained in culture in RPMI 1640 medium (Invitrogen, Paisley, United Kingdom) supplemented with 10% human serum and buffered with 25 mM HEPES (Sigma-Aldrich, St Louis, MI, USA) and 25 mM NaCO_3 _(Sigma) and in an atmosphere of 5% O_2_, 5% CO_2_, and 90% N_2 _[14]. The haematocrit was maintained at 6% and the parasitaemia at 3-5%. Culture medium was changed every day. Strain clonality was verified every month using PCR genotyping of polymorphic genetic markers (*msp1*, *msp2 *and microsatellite loci) [15,16].

### *Plasmodium falciparum *culture synchronization

To obtain tightly synchronized parasite cultures, several synchronization steps were successively employed. First, parasitized erythrocytes were treated with D-sorbitol (ICN Biomedicals, Inc., CA, United States of America) as previously described [17]. This step enriched cultures in the ring parasite stage. Secondly, in the next parasitic cycle, schizonts were selected using CS columns on a VarioMACS unit (Miltenyi Biotec, Germany) according to standard procedures [18]. Five hours later, the culture was treated with 5% D-sorbitol to eliminate parasites in mature stages (schizonts). These successive parasite synchronization steps allowed us to obtain parasites tightly synchronized in the ring stage (the window from 0 to 5 h after parasite invasion of the erythrocyte). The synchronized parasites were maintained in standard culture conditions before treatment.

### Hyperoxia exposure of *P. falciparum *cultures

Tightly synchronized cultures (ring stage aged between 4-9 hours) were split and subjected to two different conditions, either a normoxic atmosphere (5% O_2_, 5% CO_2_, 90% N_2 _gas mixture) or a hyperoxic atmosphere (21% O_2_, 5% CO_2_, 74% N_2 _gas mixture) at 37°C in two series II incubators (Model 3131, Forma Scientific, Inc.). Culture conditions were maintained over eight hours for transcript analysis (parasites aged between 12-17 hours) and for 12 hours for protein analysis (parasites aged between 16-21 hours). For each culture condition, four biological replicates were performed. Viability, parasitaemia and erythrocytic cycle stage proportions were monitored daily by examining blood smears stained with RAL^® ^555 (RAL, Martillac, France). Blood smears were taken at 0, 24, 32, 36, 48, and 78 hours.

### RNA extraction

After incubation under normoxic or hyperoxic atmospheric conditions, total RNA from parasitized erythrocytes was extracted with TRIzol reagent (Invitrogen) according to the manufacturer's instructions. RNA extracts were treated with 1 U of RNase-free DNase I (Applied Biosystems, CA, United States of America) and quantified using a NanoDrop ND-1000 (Nanodrop Technologies, Wilmington, United States of America). The integrity of the RNA was controlled with an RNA nano chip (2100 Bioanalyzer, Agilent Biotechnologies, Wilmington, DE). Samples were immediately used or stored at - 80°C.

### Microarray experiments and analysis

A 2X11 k custom *P. falciparum *whole genome microarray was designed and manufactured using SurePrint Inkjet technology^® ^(Agilent Technologies). In brief, the microarray was composed of 10,128 sixty-mer oligonucleotides representing 5,364 coding sequences located in the chromosomal, apicoplastic and mitochondrial genomes. Additional probes were added to control for quality. Labelling and hybridization was carried out following the manufacturer's protocol (Two-Color Microarray-Based Gene Expression Analysis, Agilent Technologies). Briefly, starting with 300 ng of total RNA, fluorescent cRNA (antisense) was generated using the Low RNA Input Fluorescent Amplification Kit (Agilent Technologies) and either cyanine 3-labeled CTP (Cy-3) or cyanine 5-labeled CTP (Cy-5) fluorescent dyes (PerkinElmer Life Sciences, Boston, MA). Dye swap hybridization was performed for 17 h at 60°C using the *In situ *Hybridization Kit Plus (Agilent Technologies). All processing steps were performed in an ozone-controlled environment ([O_3_] < 2 ppb) to avoid ozone-induced degradation of cyanine dyes on microarray slides. Slides were scanned at 5 μm resolution with a G2505B DNA microarray scanner (Agilent Technologies). Image analysis and intra-array signal correction was performed using the Agilent Feature Extractor Software A.9.1.3. Data processing, analysis and visualization were performed using the Resolver software 7.1 (Rosetta Inpharmatics). An error model-based transformation pipeline was used to map replicate reporters to genes, perform inter-array normalization and calculate fold changes (FC) as described elsewhere [19]. Using these FC values, a gene set enrichment analysis (GSEA) was performed using the Mann-Whitney-U-test enrichment algorithm in the PathwayStudio software 6.0 (PS 6.0™, Ariadne Genomics). The GSEA procedure determines whether the behaviour of an *a priori *set of genes shows significant concordance across two different biological states. This GSEA analysis focuses on groups of genes that share common biological function in revealing differential levels of each transcript. A gene network was generated based on information extracted from the literature using Medscan™ and the *P. falciparum*-specific database PS 6.0™ and using the "physical or regulatory connections" parameter between genes. Fold change values of microarray data were imported into PS 6.0™ and used to interpret the pathway with gene regulation networks.

### Real-time quantitative RT-PCR

cDNA was synthesized from total RNA (DNA-free) with random hexamers using the High-Capacity cDNA Archive Kit (Applied Biosystems). Primers with a melting temperature between 55 to 60°C were designed to yield a 94- to 146-bp product preferentially containing an exon/intron boundary. Specific primer sequences used for the qRT-PCR are summarized in Additional data (Additional file 1). Real-time PCR was performed using the 7900 HT Fast Real-Time PCR System (Applied Biosystems) in a 25-μL reaction volume with the Power SYBR Green^® ^PCR Master Mix Kit (Applied Biosystems). Each sample was assayed in triplicate and analysed with the ABI PRISM Sequence Detection System software Version SDS 2.2.1 (Applied Biosystems). Amplification of the *18S rRNA *sequence served as the internal control for normalization. At the end of each reaction, cycle threshold (Ct) was manually set to the level that reflected the best kinetic PCR parameters, and melting curves were acquired and analysed. Relative quantification analysis was performed using the 2-ΔΔCt method where ΔΔCt = (Ct*_target _*- Ct*_18S _*_rRNA_)_H_- (Ct*_target _*- Ct*_18S __rRNA_*)_N_., and the data reflect changes in target gene expression between two experimental conditions (N: normoxic and H: hyperoxic groups) [20].

### Protein sample preparation and CyDye labelling

After incubation under normoxic or hyperoxic atmospheric conditions, parasitized erythrocytes were washed three times in PBS medium (Invitrogen) and lysed in cold H_2_O-saponin (0.1%, Sigma) for 10 min. The lysate was then centrifuged at 1500 g for 5 min. The supernatant was discarded and the pellet containing free parasites was recovered by washing in PBS medium followed by a centrifugation step (1500 g for 5 min). The free parasites were washed until the supernatant became colourless. The pellet was then suspended in 4% (w/v) CHAPS (Sigma) and disrupted by ultrasonication (Vibracell 72412, Bioblock Scientific, Illkirch, France) five times for 60 seconds on ice at maximum amplitude. The lysate was then centrifuged at 16000 g for 15 min. The supernatant was further precipitated with acetone 100% (Sigma). The protein concentration for each sample was estimated using the BioRad Lowry-based DC assay (Biorad, Hercules, CA, USA) according to the manufacturer's instructions. Total proteins were suspended in standard cell lysis buffer (7 M urea, 2 M thiourea, 4% CHAPS, 30 mM Tris base, pH 8.5 (Sigma)) to obtain a protein concentration adjusted to 2.5 μg/μL. Protein samples were minimally labelled with CyDye according to the manufacturer's protocols (GE Healthcare, Piscataway, NJ) [21]. The mixture of labelled proteins was then separated by two-dimensional (2D) electrophoresis.

### Two-dimensional electrophoresis, image analysis and in-gel digestion

Isoelectric focusing (IEF) was performed on 18-cm 3-10 linear IPG strips (GE Healthcare). Destreak buffer containing 1% (v/v) IPG buffer 3-10 was used for overnight rehydration of IPG strips. The samples were applied at the acidic end of the IPG strip using a cup-loading technique. IEF was carried out on a Ettan IPGphor II (GE Healthcare) electrophoresis unit at 20°C for a total of 45 kVh (ramp to 300 V in 3 hrs, ramp to 1000 V in 6 hrs, ramp to 8000 V in 3 hrs, hold at 8000 V for 4 hours). IPG strips were equilibrated in equilibration buffer containing 50 mM Tris-HCl, pH 8.6, 6 M urea, and 2% SDS and 30% glycerol supplemented with 1% (w/v) DTT for 15 min at room temperature, followed by protein alkylation (carbamidomethylation) in the same equilibration buffer containing 2.5% (w/v) iodoacetamide instead of DTT for 15 min at room temperature. IPG strips were then placed on the top of 10% uniform polyacrylamide gels. Strips were overlaid with 0.5% agarose in 1× running buffer containing bromophenol blue and the proteins were further separated by SDS-PAGE (10 W per gel) at 20°C in an Ettan DALT Six electrophoresis system (GE Healthcare). After electrophoresis, the gels with Cydye-labelled proteins were directly imaged using a Typhoon™ Trio Image scanner (GE Healthcare UK). The intensity was adjusted to ensure that the maximum volume of each image was within 60,000 - 80,000 U. Analysis of 2-D DIGE was performed with DeCyder 6.5 software (GE Healthcare) using the differential in-gel analysis (DIA) and the biological variation analysis (BVA) modules. Protein spots that were expressed differentially between two experimental conditions (|ratio|≥1.5, *p *≤ 0.05 *t-Test*) were marked with master gel numbers. Based on DeCyder v6.5 analysis, spots of interest from gels stained with Imperial Blue Stain (Pierce) were excised and digested using a Shimadzu Xcise automated gel processing platform (Shimadzu Biotech, Kyoto, Japan) as described previously [22] and stored at -20°C.

### Mass spectrometry analysis

The samples were analysed by nanoscale capillary liquid chromatography-tandem mass spectrometry (nano LC-MS/MS). Purification and analysis were performed on a C18 capillary column using a CapLC system (Waters, Milford, MA) coupled to a hybrid quadrupole orthogonal acceleration time-of-flight tandem mass spectrometer (Q-TOF Ultima, Waters, MA). Chromatographic separation was conducted on a reversed-phased capillary column (Atlantis™ dC18, 3 μm, 75 μm × 150 mm Nano Ease™, Waters, MA) with a 180-200 nl min^-1 ^flow. The gradient profile consisted of a linear gradient from 95% A (H_2_O, 0.1% HCOOH) to 60% B (80% ACN, 0.1% HCOOH) in 60 min followed by a linear gradient to 95% B in 10 min. Mass data acquisitions were piloted by MassLynx 4.0 software using automatic switching between MS and MS/MS modes. The internal parameters of Q-TOF were set as follows. The electro-spray capillary voltage was set to 3.2 kV, the cone voltage was set to 30 V, and the source temperature was set to 80°C. The MS survey scan was m/z 400-1300 with a scan time of 1 s and an interscan time of 0.1 s. When the intensity of a peak rose above a threshold of 15 counts, tandem mass spectra were acquired. Normalized collision energies for peptide fragmentation were set using the charge-state recognition files for +2 and +3 peptide ions. The scan range for MS/MS acquisition was from m/z 50 to 1500 with a scan time of 1 s and an interscan time of 0.1 s. Fragmentation was performed using argon as the collision gas and with the collision energy profile optimized for various mass ranges and charges of precursor ions. Mass data collected during a nano LC-MS/MS analysis were processed using ProteinLynx Global Server 2.2 software (Waters) with the following parameters: no background subtraction, smooth 3/2 Savitzky Golay and no deisotoping to generate peak lists in the micromass pkl format. Pkl files were then fed into the local search engine Mascot Daemon v2.2.2 (Matrix Science, London, UK). The data were searched against the *Homo sapiens *(218356 sequences) and *P. falciparum *(13110 sequences) National Center for Biotechnology Information non-redundant (NCBInr) protein database (March, 2010). Search parameters allowed for one missed tryptic cleavage site, the carbamidomethylation of cysteine, and the possible oxidation of methionine; precursor and product ion mass error tolerance was < 0.2 Da. All identified proteins had a Mascot score greater than 29 and 38 respectively for *P. falciparum *and *Homo sapiens*, corresponding to statistically significant (*p *≤ 0.05 *t-Test*) identification. Identifications were considered valid when they contained at least two peptide sequences per protein. If a single peptide sequence was identified per one protein, the mascot score and sequence coverage were taken into account (Additional file 2).

## Results

### Effect of hyperoxia on *P. falciparum *and experimental design

**Figure 1:**
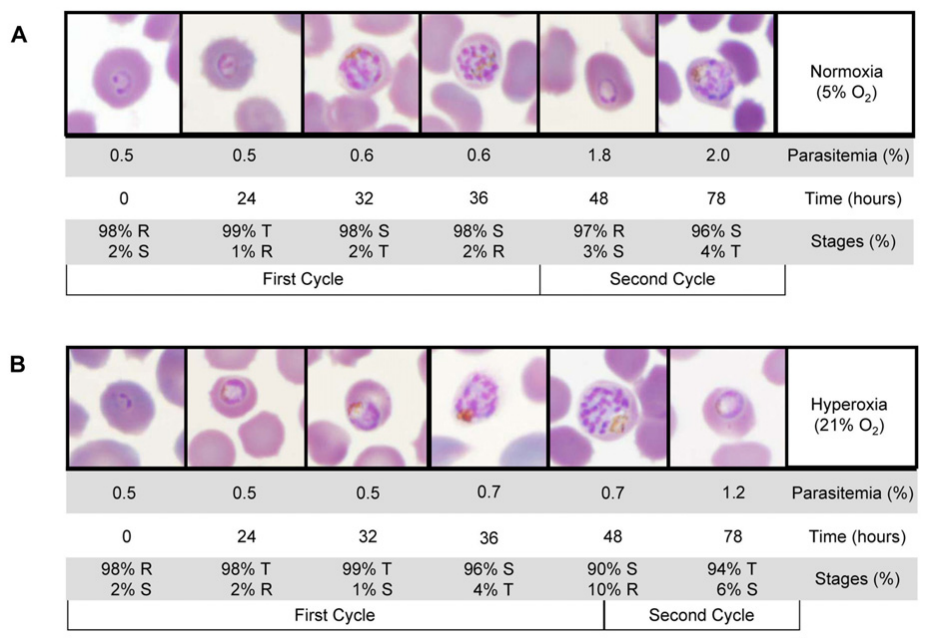
**Lengthening of *P. falciparum *cell cycle following hyperoxia exposure**. Phenotypic changes in *P. falciparum *asexual blood stages were observed during two cycles under normoxic (A) and hyperoxic (B) conditions. The parasitaemia and parasitic stages were evaluated by blood smears at different times: 0, 24, 32, 36, 48, and 78 hours. The different intraerythrocytic stages and their corresponding percentages are indicated as follows: ring (R), trophozoite (T) and schizont (S).

To determine the effects of hyperoxia on asexual blood stage *P. falciparum *parasites, tightly synchronized cultures of the 3D7 strain were exposed to normoxic (5% O_2_) and hyperoxic (21% O_2_) conditions for two life cycles. The proportion of parasitaemia and erythrocytic cycle stages were monitored by blood smears in triplicate at different times: 0, 24, 32, 36, 48, and 78 hours (Figure 1). In normoxic condition, the 3D7 *P. falciparum *strain had a life cycle of 45 hours with entry into schizogony at approximately 32 hours. Parasite exposure to 21% O2 increased the length of parasitic cycle and decreased the parasitaemia, but it did not alter the parasites morphology (Figure 1). At 48 hours, the second parasitic cycle began for parasites exposed to 5% O_2 _(nearly all of them were at ring stage (100%)), while the majority of parasites exposed to 21% O_2 _remained in the schizont stage (90% schizonts and 10% ring). Thus, hyperoxia induces a delay of *P. falciparum *cell cycle of four hours as previously described [23]. After reinvasion during the following cycle (third cycle), hyperoxia exposure did not change parasitaemia and parasites had a normal life cycle without excess lethality (unpublished data). These results seem to indicate a biological adaptation of the parasite to hyperoxia. To study the effects of hyperoxia on *P. falciparum*, complementary high-throughput transcriptomic and proteomic approaches were used. Transcriptome and proteome profiles from parasitized RBCs exposed to normoxic (5% O_2_) or hyperoxic (21% O_2_) atmospheric conditions were compared. The results were controlled by the experimental design on two levels: (i) the percentage of atmospheric oxygen and (ii) the timing of the parasitic stage. First, hyperoxic exposure was chosen at 21% O_2 _in light of the above data [23]. Second, transcriptome and proteome experiments were performed at the late-ring stage, after RBC reinvasion with a synchronization window of four hours. This stage allowed us to avoid the effects of cycle delay.

### *Plasmodium falciparum *response to hyperoxia treatment: microarray analysis

To investigate the response of *P. falciparum *to hyperoxia, the 3D7 strain was cultured *in vitro *under normoxic (5% O_2_) and hyperoxic (21% O_2_) atmospheric conditions. Three biological replicates in each group were performed and comparisons were made with a dye-swap experimental scheme. The raw microarray data are available in the Gene Expression Omnibus database [platform GPL9482 and samples from GSM466802 to GSM466807, 2010 [24]]. Among 5,364 coding sequences represented on the microarray, 219 genes were significantly altered following hyperoxia exposure (*p *≤ 0.01, Student's *t-Test*, |FC|≤ 1.5, Additional file 3), among which 114 were up-expressed and 105 were down-expressed. Based on the selected 219 genes, a GSEA (FDR ≤ 0.05) was performed [25], and this allowed us to define 9 functional groups that were significantly altered (following hyperoxia treatment) (Additional file 4 and Table 1). Among these functions, "DNA repair," "Vacuolar acidification" and "Response to oxidative stress" were previously reported to be involved in the hyperoxia response [10,26-30].

**Table 1:** Biological functions perturbed following hyperoxia exposure on *P. falciparum*

Functional group^a^	Number of entities^b^	*p*-value^c^
GPI anchor biosynthesis	29	0.0006
DNA repair	15	0.0072
Vacuolar acidification	11	0.0255
Actin filament organization	12	0.0296
Nucleosome assembly	12	0.0490
Regulation of cell shape	6	0.0519
Leading strand elongation	10	0.0533
Lysosomal H+ import	26	0.0589
Response to oxidative stress	17	0.0799

^a^Gene Set Enrichment Analysis (GSEA) using the Mann-Whitney-U-test enrichment algorithm in PathwayStudio software indicated functional groups significantly altered (*p *< 0.08). For each functional group, the ^b^number of genes included and corresponding ^c^*p*-values are listed.

**Figure 2:**
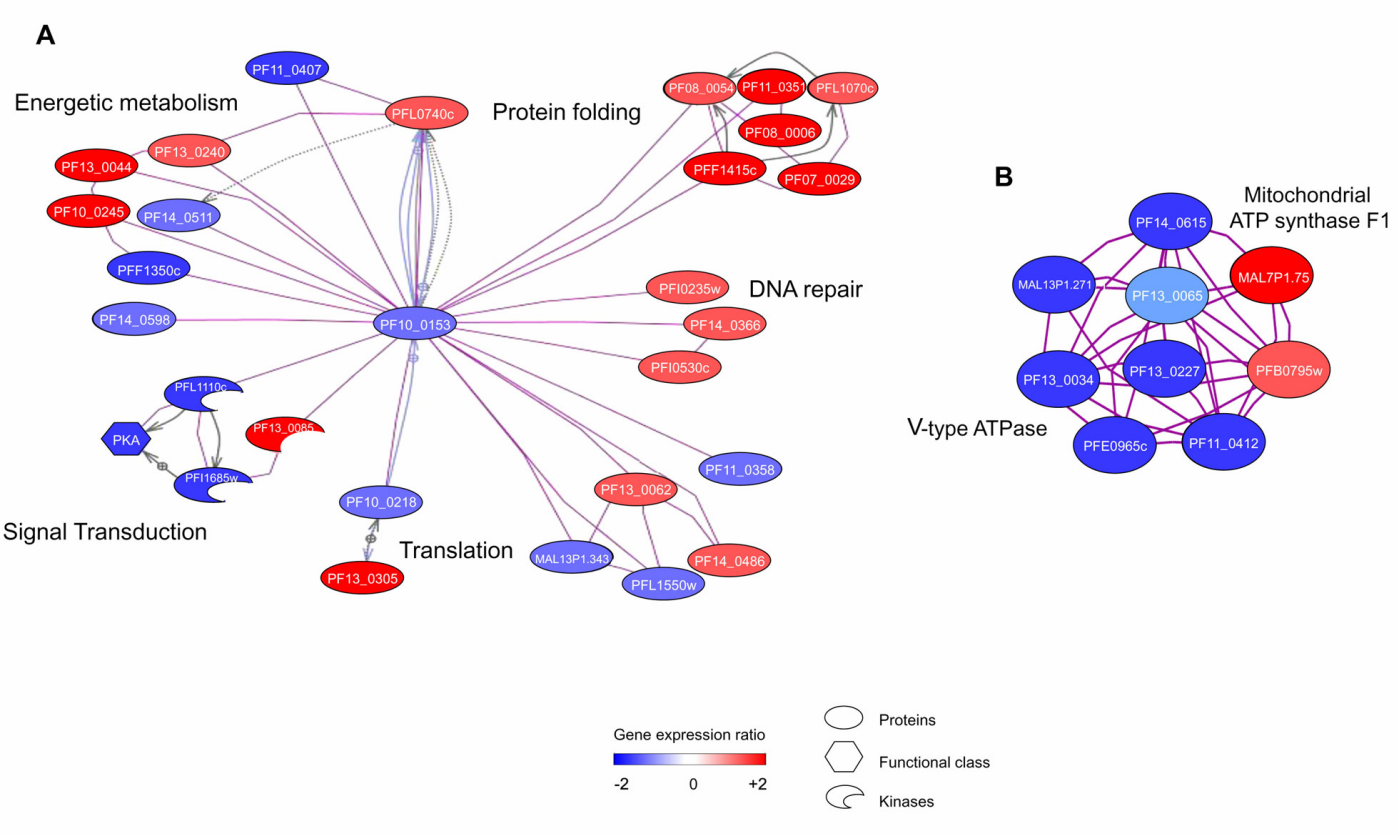
**Gene networks involved in the metabolic adaptation to hyperoxia in tightly synchronized *P. falciparum *cultures**. Using PS 6.0™ software and microarray expression data, gene networks were built and genes modulated in response to hyperoxia are represented. (A) Representation of metabolic interrelations related to adaptative hyperoxia exposure. *Energetic metabolism: aspartate carbamoyltransferase *(P13_0240), *carbamoyl-phosphate synthetase *(PF13_0044), *glutamine-fructose-6-phosphate transaminase *(PF10_0245), *glucose-6-phosphate dehydrogenase *(PF14_0511), *acetyl-CoA synthetase *(PFF1350c), *gapdh *(PF14_0598) - *Signal transduction: regulatory sub-unit of cAMP-dependent protein kinase *(PFL1110c), *catalytic sub-unit of cAMP-dependent protein kinase *(PFI1685w), *ser thr protein kinase *(PF13_0085) - *Translation: citrate synthase *(PF10_0218), *translation elongation factor 1 alpha 1 *(PF13_0305), *proteasome 26S subunit *(MAL13P1.343), *dihydrolipoamide dehydrogenase *(PFL1550w), *translation elongation factor 2 *(PF14_0486), *adaptor-related protein complex 1 *(PF13_0062), *polymerase RNA I *(PF11_0358) - *DNA repair: DNA primase *(PF14_0366 and PFI0530c), *rpa1 *(PFI0235w) - *Protein folding: ferredoxin reductase *(PF11_0407), *Hsp10 *(PFL0740c), *Hsp60 *(PF10_0153), *Hsp70 *(PF11_0351 and PF08_0054), *Hsp90 *(PFL1070c and PF07_0029), *prohibitin *(PF08_0006), *DnaJ *(PFF1415c). (B) Representation of ATP-dependent gene sub-networks altered in hyperoxic conditions. *V-type ATPase: V-type ATPase putative *(MAL13P1.271), *vacuolar ATP synthase subunit h putative *(PF13_0034), *vacuolar ATP synthetase putative *(PFE0965c), *vacuolar ATP synthase subunit F putative *(PF11_0412), *vacuolar ATP synthase subunit D putative *(PF13_0227), *vacuolar ATP synthase catalytic subunit a *(PF13_0065) - *Mitochondrial ATP synthase F1: ATP synthase subunit putative *(PF14_0615), *mitochondrial ATP synthase F1 epsilon subunit *(MAL7P1.75), *mitochondrial ATP synthase F1 alpha subunit putative *(PFB0795w). Red and blue colors correspond respectively to up- and down-regulated genes compared between hyperoxic to normoxic conditions.

GSEA data were first integrated to create a gene network based on information extracted from the literature using Medscan™ and PS 6.0™ (Ariadne Genomics). Next, the expression levels of genes included in the gene network were assigned using PS 6.0™ and microarray expression data. This programme gives a dynamic view of metabolism during the hyperoxia response (Figures 2A and 2B). Thus, PS 6.0 analysis clustered 28 modulated-expression genes in five metabolic groups labelled "Energetic metabolism," "Protein folding," "Signal Transduction," "DNA repair" and "Translation" (Figure 2A). This transcriptomic analysis allowed us to identify up-regulated genes involved in DNA repair and protein folding and down-regulated genes linked to PKA-dependent signal transduction and glycolysis (Figure 2A). Additionally, PS 6.0 software revealed an alteration of an ATP-dependent sub-network: specifically, up-regulation of the mitochondrial ATP synthase complex and down-regulation of the V-type ATPase complex (Figure 2B).

**Figure 3:**
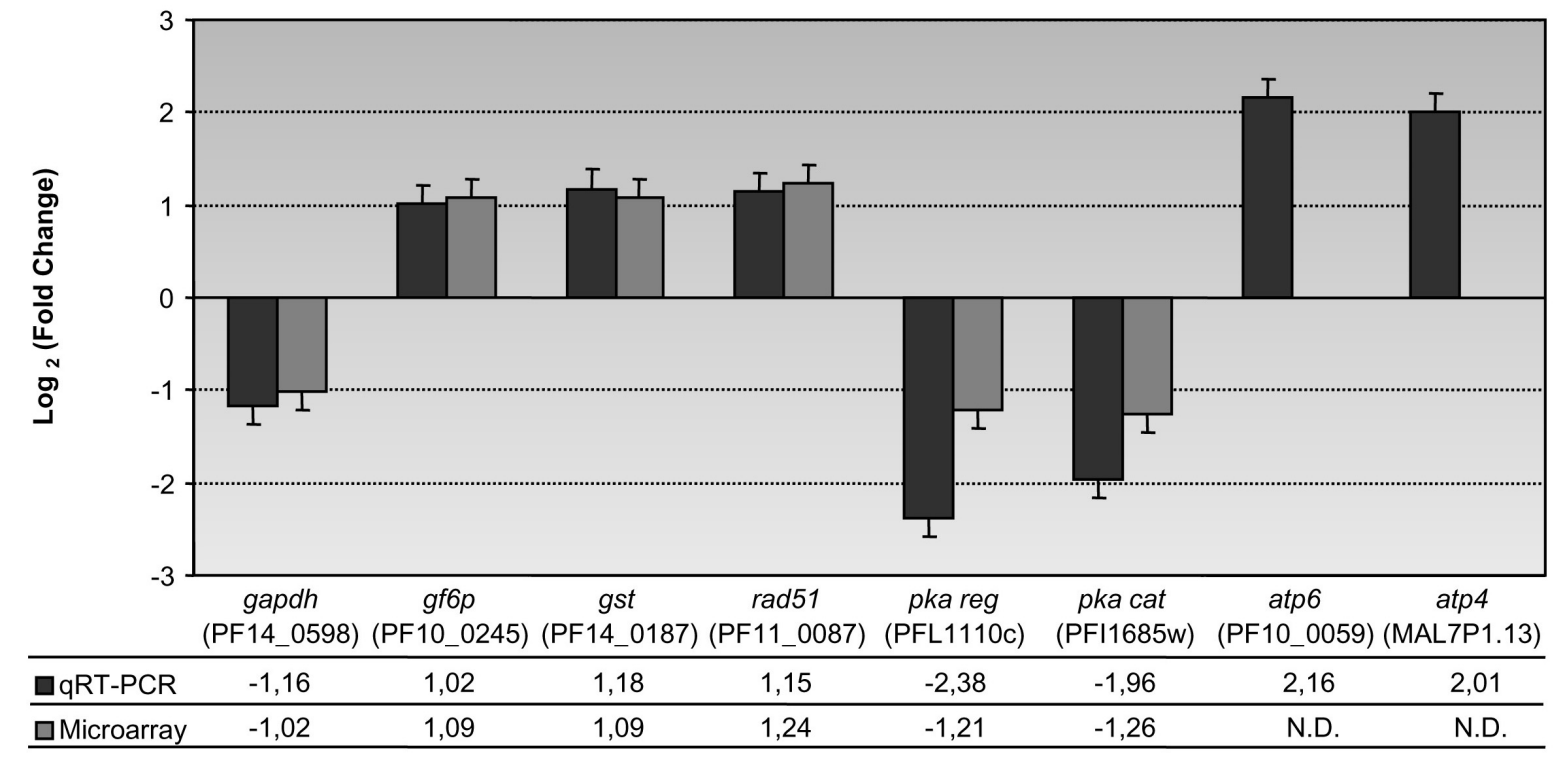
**Validation of microarray data by qRT-PCR**. Data from qRT-PCR and a microarray of eight selected genes were compared between hyperoxic and normoxic conditions. Adjacent bars correspond to the mean log_2 _(fold change) and respective standard deviation and present qRT-PCR and microarray results in gray scale for the respective gene. The abbreviations and their corresponding gene IDs (PlasmoDB accession numbers) are indicated below the graphic. Crude values of mean fold changes are presented in the table. The same samples were used for the qRT-PCR and microarray experiments. *gapdh*: glyceraldehyde-3-phosphate dehydrogenase - *gf6p*: glutamine-fructose-6-phosphate transaminase - *gst*: glutathione S-transferase - *pka*: protein kinase A - *atp6*: mitochondrial ATP synthase F_0 _a subunit - *atp4*: mitochondrial ATP synthase F_0 _b subunit.

To confirm the GSEA data, eight genes presenting significant variations in expression were quantified using real-time qRT-PCR (Additional file 1). These genes, which were involved in glycolysis [PlasmoDB: *PF14_0598, PF10_0245*], antioxidant metabolism [PlasmoDB: *PF14_0187, PF11_0087*], signal transduction [PlasmoDB: *PFL1110c, PFI1685w*] and ATP synthase activity [PlasmoDB: *PF10_0059, MAL7P1.13*] were chosen according to their essential functions in response to hyperoxia. qRT-PCR was performed on total RNA extracted from the same three samples used for microarray analysis. Correlation coefficients for each specific-gene standard curve were always > 0.99 (unpublished data). Gene amplification was normalized by *18S rRNA *[PlasmoDB: *MAL7_18Sa*] levels as previously described [31]. The two analyses (qRT-PCR and microarray) yielded consistent results for all the genes evaluated (Figure 3).

### *Plasmodium falciparum *response to hyperoxia treatment: proteomic analysis

**Figure 4:**
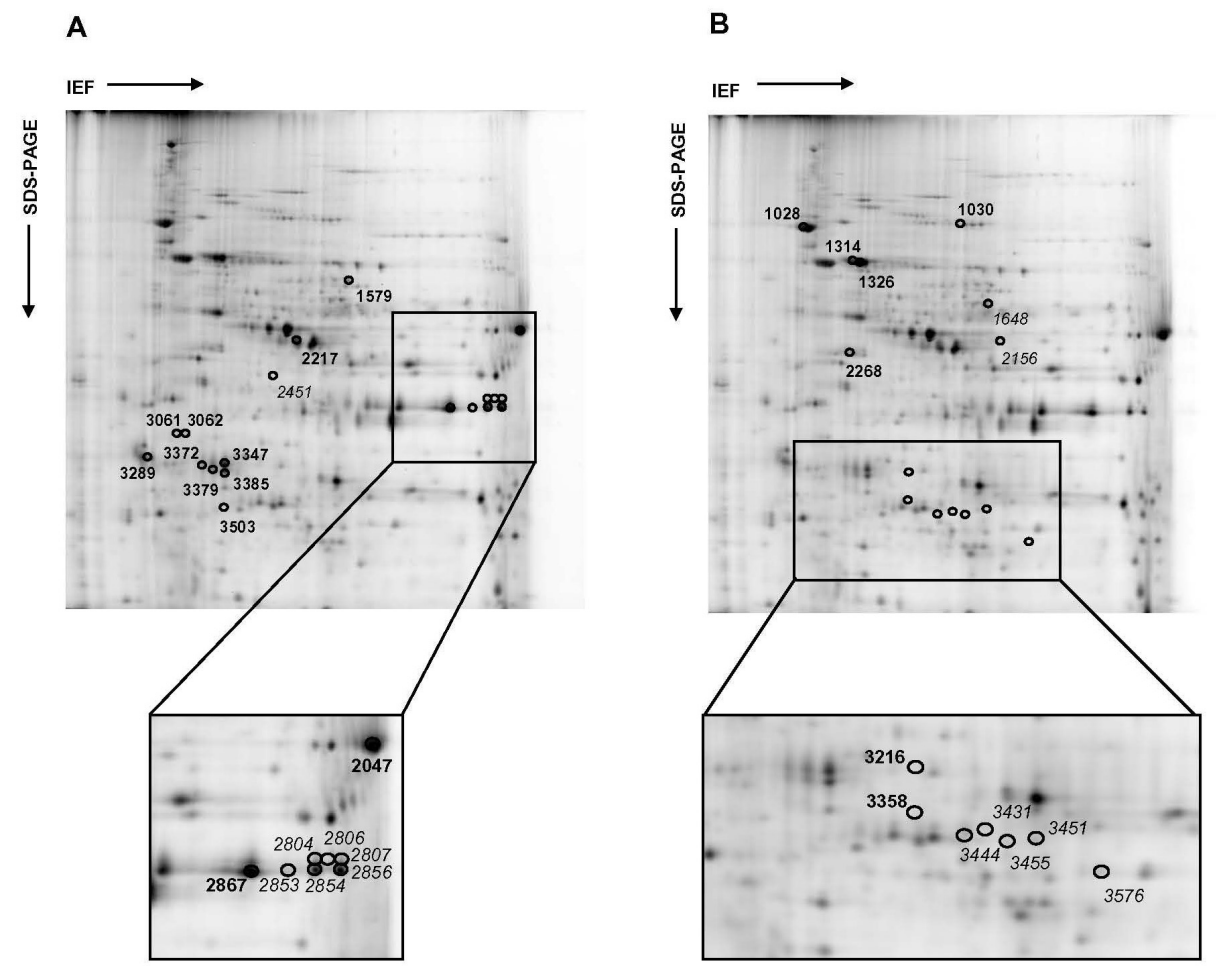
**Alterations of the *P. falciparum *proteome under hyperoxic exposure**. The proteins from *P. falciparum *parasites cultivated under normoxic (A) or hyperoxic (B) conditions were labelled with Cy3 and Cy5, respectively, and separated by 2-DE using a 10% homogeneous SDS polyacrylamide gel with a pH range from 3 to 10. As determined by DeCyder software, protein spots that were down- (A) or up- (B) regulated following hyperoxic exposure (|FC|≥1.5, *p *≤ 0.05) are marked with master numbers (Table 2 and Additional file 5). Bold and italic numbers correspond, respectively, to proteins identified from *P. falciparum *and *Homo sapiens*. Areas of gels containing a high density of spots down- and up-modulated are enlarged.

**Figure 5:**
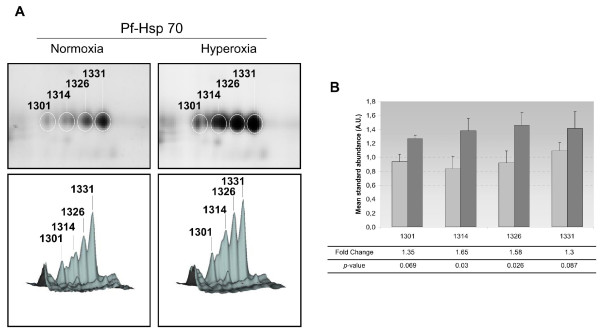
**Selective post-translational modification of Pf-Hsp70 following hyperoxia exposure**. (A) Enlarged 2D-DIGE gel images and their corresponding three-dimensional profiles are shown for a series of four protein spots identified as Pf-Hsp70. The amount of protein is proportional to the volume peak. Numbers correspond to master gel and significant deregulated spots (*i.e*., 1314 and 1326) are reported in the table 2. (B) A graphic quantification of the four spots corresponding to Pf-Hsp 70 under normoxic (light-gray bars) and hyperoxic conditions (dark-gray bars). Spot numbers are specified at the bottom. Adjacent bars correspond to the mean standard abundance and respective standard deviation. Fold change and *p*-values are indicated at the bottom for each spot. A.U.: arbitrary units, FC: fold change.

To identify *P. falciparum *proteins involved in the hyperoxia response, 2D-DIGE experiments coupled to MS were performed. Four independent cultures of *P. falciparum *cultivated under normoxic and hyperoxic conditions were included in this analysis. After protein separation on 2-DE, each gel was individually imaged and all gel images were analysed using the DeCyder 6.5 software. Among 1840 protein spots matched, 33 spots were significantly modulated (|FC|≥1.5, *p *≤ 0.05 *t-Test*) following hyperoxia treatment (14 and 19 spots were up- and down-modulated, respectively; Figure 4). All spots were successfully identified by MS and corresponded to 14 *Homo sapiens *and 19 *P. falciparum *proteins (Table 2 and Additional file 5). However, some proteins were detected in more than one spot, indicating different isoforms. Indeed, only six proteins were identified for *Homo sapiens *and 13 for *P. falciparum *(Table 2). These results indicated that hyperoxia induced protein modulations at two levels: namely, protein expression and post-translational modification. Among the four spots detected (spot numbers 1301, 1314, 1326, and 1331) as Pf-Hsp70 protein [PlasmoDB: PF08_0054, GenBank: gi|124512406], only two isoforms were significantly up-regulated in hyperoxic conditions (Table 2 and Figure 5). These results indicated that the hyperoxia response could induce post-translational regulation of several parasite proteins.

**Table 2:** Proteins identified by differential 2-D DIGE analysis following hyperoxia exposure of *P. falciparum*

gi numberc	Gene ID	Protein name	MW (kDa)	pI	Master spot number	Significance (Mascot score)	Average ratio	*t-Test*
***P. falciparum***								

**Translation**								

gi|124512420	MAL8P1.69	14-3-3 protein homologue	29.86	4.96	3289	425	-1.54	0.0078

gi|124513850	PF13_0304	elongation factor 1 alpha	49.16	9.12	2047	130	1.60	0.0090

gi|8918238	PF14_0486	elongation factor 2	85.03	6.30	1030	67	1.72	0.0070

gi|124810293	PF14_0655	RNA helicase-1	45.62	5.48	2268	105	1.53	0.040

**Parasitophorous vacuolar membrane Transporter**								

gi|124810348	PF14_0678	exported protein 2	33.62	5.10	3061	88	-1.92	0.00093

					3062	180	-2.06	0.0046

**Glycolysis**								

gi|124809201	PF14_0341	glucose-6-phosphate isomerase	67.61	6.78	1579	36	-1.63	0.00092

gi|124810131	PF14_0598	glyceraldehyde-3-phosphate dehydrogenase	37.08	7.59	2867	695	-1.69	0.033

**Chaperone-assisted protein folding**								

gi|124512406	PF08_0054	heat shock protein 70	74.39	5.51	1314	257	1.65	0.03

					1326	137	1.58	0.026

gi|505340	PF07_0029	heat shock protein 86	86.77	4.91	1028	412	1.50	0.0052

**Amino acids metabolism**								

gi|86170756	PFF0435w	ornithine aminotransferase	47	6.47	2217	96	-2.14	0.0081

gi|124513590	MAL13P1.214	phosphoethanolamine	31.31	5.43	3347	145	-2.05	0.0055

		N-methyltransferase			3372	252	-1.96	0.00089

					3379	253	-2.17	0.00058

					3503	52	-1.62	0.00056

					3385	452	-2.71	0.00026

**Proteasome-mediated proteolysis**								

gi|124512686	MAL8P1.142	proteasome beta-subunit	31.08	6.00	3216	58	1.78	0.015

gi|124513790	MAL13P1.270	proteasome subunit	27.50	6.17	3358	142	1.55	0.049

***Homo sapiens***								

**Oxygen transporter**								

gi|183817		Beta-globin	19.21	6.28	3451	165	7.71	0.020

					3576	102	1.91	0.011

**Antioxidant metabolism**								

gi|4502517		carbonic anhydrase I	28.91	6.59	3431	173	4.18	0.0095

					3444	155	3.47	0.0019

					3455	224	4.02	0.00038

gi|4557014		catalase	59.95	6.9	1648	200	6.47	0.012

gi|16306550		selenium binding protein 1	52.93	5.93	2451	73	-1.64	0.041

gi|168985379		flotillin 1	39.81	6.03	2156	157	1.64	0.040

**Glycolysis**								

gi|31645		glyceraldehyde-3-	36.20	8.26	2804	199	-1.94	0.0042

		phosphate dehydrogenase			2806	181	-1.99	0.012

					2807	132	-1.84	0.0069

					2853	112	-2.17	0.0027

					2854	122	-2.25	0.0032

					2856	96	-2.20	0.0019

The proteins were identified by mass spectrometry following in gel trypsin digestion. The spot numbers correspond to the same numbers in figure 4. The Mascot gi number of the spots, their gene ID (PlasmoDB), their name, the theoretical MW and *pI *values, as well as the corresponding Mascot score are listed for MS/MS analysis (scores greater than 29 for *P. falciparum *and 38 for *Homo sapiens *are considered significant (*p *≤ *0.05*)). Paired average volume ratio (hyperoxic versus normoxic conditions) and *p*-values (*t-Test*) were obtained using Decyder software. MW: molecular weight.

To determine the metabolic pathways perturbed following hyperoxia, the identified proteins were classified using the NCBI COG database. The proportion of modulated proteins involved in each functional category was determined as follows. For *P. falciparum*, six functional categories were found to be altered, among which were chaperone-assisted protein folding, translation, antioxidant metabolism and glycolysis, which were already identified in transcriptomic analysis. For *Homo sapiens*, the identified proteins were classified into three functional categories: antioxidant metabolism, glycolysis and O_2 _transporter (Table 2).

**Figure 6:**
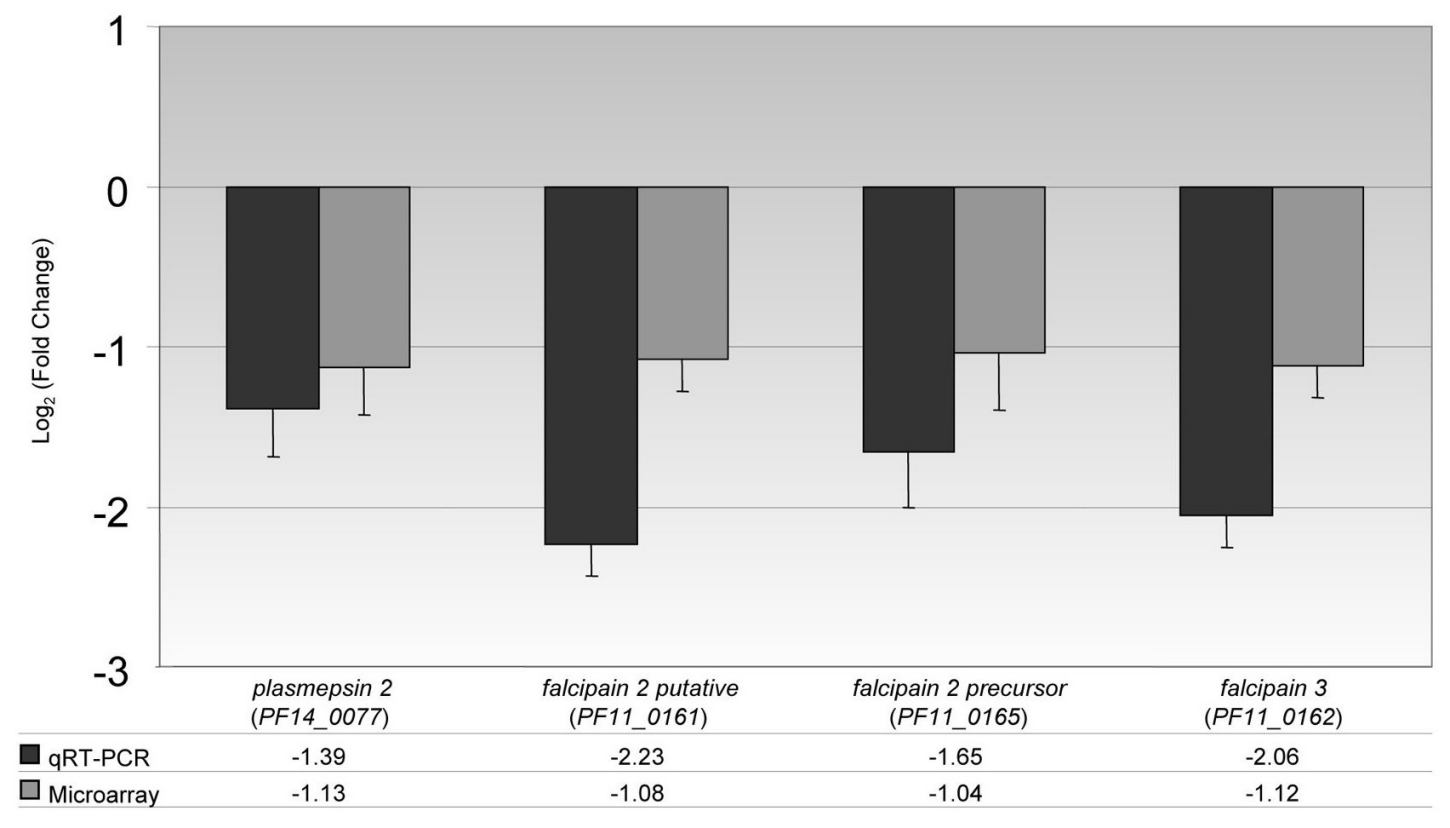
**Analysis of the beta-globin digestion pathway**. qRT-PCR and microarray data of transcripts involved in digestive vacuole metabolism were compared between hyperoxic and normoxic conditions. Adjacent bars correspond to the mean log_2 _(fold change) and respective standard deviation and present qRT-PCR and microarray results in gray scale for the respective gene. The abbreviated names and their corresponding gene ID (PlasmoDB accession numbers) are indicated below the graphic. Crude values of mean fold changes are presented in the table. The same samples were used for the qRT-PCR and microarray experiments.

The 2D-DIGE analyses also indicated the accumulation of some proteins involved in digestive vacuole metabolism such as human catalase [GenBank: gi|4557014] and beta-globin [GenBank: gi|183817]. The accumulation of beta-globin suggests proteases inhibition of the beta-globin degradation pathway. To explore this hypothesis, the transcripts of four genes [PlasmoDB: *PF14_0077, PF11_0161, PF11_0165, PF11_0162*] were quantified using real-time qRT-PCR as described above (Additional file 1). All genes involved in digestive vacuole metabolism were found to be down-expressed (Figure 6).

## Discussion

*In vivo*, the *P. falciparum *parasite is subjected to varying oxygen levels throughout its life cycle (*i.e*., from 5% O_2 _in human venous blood to 13% O_2 _in the human lungs and 21% O_2 _in mosquito salivary glands). In the mosquito, the metabolic adaptation of parasite to oxygen-rich environment involved mitochondrial and physiological differences [32]. These oxygen variations imply that metabolic adaptation of *P. falciparum *is crucial for it survival. During malaria complications such as acute respiratory distress syndrome, late-ring stage parasites are susceptible to sequestration in pulmonary capillaries and are thus exposed to hyperoxic conditions [33]. These sequestered young parasites could be exposed to higher oxygen levels than the physiologically relevant O_2 _tension. Additionally, Blanco *et al *reported that hyperbaric oxygen therapy (HBO, 100% O_2_) has a beneficial effect on malaria syndrome evolution [34]. Indeed, a better understanding of the metabolic adaptation of the malaria parasite to hyperoxia could help to develop new anti-malarial drug treatments that could be used in association with HBO treatment. To study the global response of *P. falciparum *to hyperoxia, a dual high-throughput approach combining microarray and 2D-DIGE analysis was used on parasite cultures under hyperoxic conditions (*e.g*., 21% O_2_). Accordingly Hsp90, Hsp70, GAPDH, and elongation factor 1 and 2 were found to be altered at the transcript and protein levels under hyperoxia.

Since the development of high-throughput technologies, few studies have been published regarding the transcriptome and proteome of *P. falciparum *in response to environmental constraints or drug treatments [35-40]. Since hyperoxia induces a *P. falciparum *cycle delay, the sample collections were performed before the phenotypic effect. Although the majority of genes had a periodic expression profile [41], RNA transcription is maximal between 18 and 24 hours of the parasitic cycle [42]. In the present study, sample collections were performed at the late-ring parasite stage. Moreover, as a delay exists between mRNA and protein accumulation [43-45], the time of exposure was also taken into account and a four hours delay was chosen between mRNA and protein sampling. It has been suggested that there may be a discrepancy between *P. falciparum *transcriptomic and proteomic responses [35,39]. Preliminary microarray experiments were performed with transcripts from synchronized *P. falciparum *in the same experimental scheme, but the samples were collected after four hours of hyperoxia treatment. These microarray analyses indicated that 176 transcripts were significantly deregulated (|FC|≤ 1.5, *p *≤ 0.01, Student's *t-Test*), and some genes significantly deregulated were involved in the early antioxidant response, such as 1-cys peroxidoxin [PlasmoDB: PF08_0131], Fe-superoxide dismutase [PlasmoDB: PF08_0071] and thioredoxin peroxidase [PlasmoDB: MAL7P1.159] (Additional file 6). This early stress response is generally observed in stress condition does not reflect a specific hyperoxia adaptation [30,46]. Here, despite significant transcript variations (*p *≤ 0.01, Student's *t-Test*), gene fold-changes observed were low (|FC|≤ 1.5) under hyperoxia. The low-level changes observed in the *P. falciparum *transcriptome could be explained by tight gene regulation [47,48] or by post-transcriptional regulation of most *P. falciparum *genes [43-45]. Consequently, analysis of the parasite's adaptive response to hyperoxia requires the use of extremely successful bioinformatic tools for microarray data interpretation such as PS 6.0 software [44,49], and this analysis was completed using a highly sensitive proteomic approach such as 2D-DIGE.

It is generally accepted that one of the first effects of hyperoxia is ROS overproduction (superoxide anions (O^-^_2_), hydrogen peroxide (H_2_O_2_) and hydroxyl radicals (OH^-^)), which is generated by metabolism, and particularly, by respiratory metabolism [50]. DNA, lipid, and protein alterations by ROS may be lethal to malaria parasites. Thus, to fight oxidative stress, *P. falciparum *has developed an adaptive defence response including repair mechanisms for nucleic acids and proteins [29,51]. Despite high ROS defence system expression under normoxia, transcriptomic analysis suggests an up-regulation of ROS defence systems [29]. Five genes involved in DNA repair were found to be up-regulated during hyperoxia. Among them, two sub-unities of *DNA primase *[PlasmoDB: *PF14_0366 *and *PFI0530c*] and *replication protein A1 *[PlasmoDB: *rpa1*, *PFI0235w*] have been described to be involved in chromosomal replication [52,53]. *Rpa1 *was reported also to interact with *rad51 *[PlasmoDB: *PF11_0087*] in nucleosomes during replication to correct DNA mismatches [51,54]. And *Hsp40 *[PlasmoDB: DNAJ homologue, *PFF1415c*] is associated with DNA repair and the replication machinery [55]. These observations suggest that DNA repair enzymes maintain the integrity of the parasitic genome under high oxygen pressure.

Protein oxidation caused by ROS is circumvented by diverse functions such as regulation of the redox state and modulation of protein stability [56]. Chaperone proteins, Hsps known as stress response proteins, further assure this protection. Several proteins involved in chaperone activity including Hsp40 [PlasmoDB: DNAJ homologue; *PFF1415c*], Hsp60 [PlasmoDB: *PF10_0153*], Hsp70 [PlasmoDB: *PF11_0351 *and PF08_0054], Hsp90 [PlasmoDB: *PFL1070c *and PF07_0029], and protein 14-3-3 [PlasmoDB: MAL8P1.69] were found up-regulated under hyperoxia. As described by Akide-Ndunge *et al *[57], *Hsp60 *is up-regulated under oxidative stress like hyperoxia and its expression is coordinated with antioxidant enzymes in a stage-dependent manner, suggesting thus that Hsp up-regulation is implicated in ROS removal. Elsewhere, Pf-Hsp70 forms a functional network in the mitochondrial matrix with DNAJ, Hsp60 and prohibitin to be involved in post-translational modification of proteins [56]. Isoforms of Hsp70 were detected following hyperoxia exposure, which may also correspond to post-translational modification as previously predicted [58,59]. These Hsps, which act as sensors of environmental conditions, are involved in adaptation mechanism by post-translational modification [56]. However, the role of these post-translational modifications on regulation of protein expression in *P. falciparum *is little known [38,39]. Nevertheless, the chaperone activity of Hsps seems regulate during the hyperoxia response and facilitate *P. falciparum *adaptation to hyperoxic environments.

**Figure 7:**
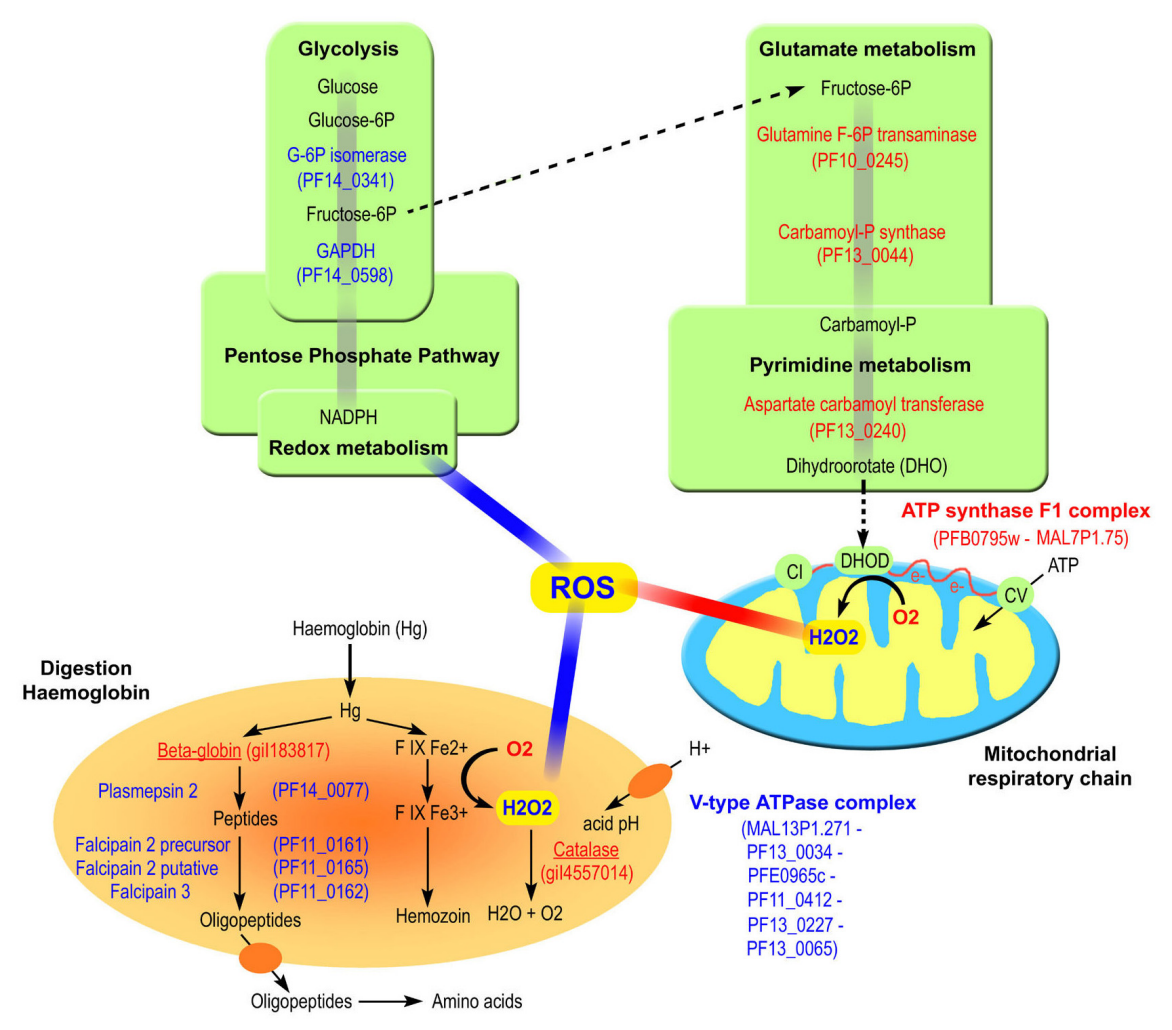
**A schematic representation of *P. falciparum *metabolic adaptation to hyperoxia exposure**. Metabolic pathways based on the Ginsburg Pathway [72] for glycolysis (cytoplasmic), respiratory chain (mitochondria, blue and yellow) and haemoglobin digestion (digestive vacuolar, orange) are shown. The gene IDs (PlasmoDB) are reported for each enzymatic reaction. Human protein names are underlined. Up- and down-regulated genes and reactive oxygen species (ROS) production are indicated in red and blue characters, respectively.

Under hyperoxia, down-regulation of glycolytic enzymes (glucose-6-phosphate isomerase [PlasmoDB: PF14_0341] and glyceraldehyde-3-phosphate dehydrogenase [PlasmoDB: GAPDH, PF14_0598]) was detected in this study. Additionally, three enzyme involved in *de novo *pyrimidine biosynthesis were found up-regulated such as carbonic anhydrase [GenBank: gi|4502517], *carbamoyl-phosphate synthetase *[PlasmoDB: *PF13_0044*] and *aspartate carbamoyltransferase *[PlasmoDB: *PF13_0240*] [60]. This last, up-expressed in microarray analysis, produces dihydroorotate oxidase (DHO), an essential substrate of mitochondrial respiratory chain complex II [61]. This pyrimidine pathway is essential for nucleic acid synthesis to repair DNA lesions caused by the oxidative stress. Microarray data indicated that *alpha and epsilon ATP synthase F1 subunits *[PlasmoDB: *PFB0795w *and *MAL7P1.75*] were up-regulated, indicating that parasites seem to produce ATP through mitochondrial respiration. Mitochondrial F_0_F_1 _ATP synthase is composed of two subcomplexes, F_0 _and F_1. _F_1 _is composed of five subunits, and these have been reported in the *P. falciparum *genome [62]. Recently, Mogi and Kita have identified four F_0 _subunits of *P. falciparum *ATP synthase [63,64] and Mather *et al. *support the existence of ATP synthase activity [65]. Two transcripts of ATP synthase F_0 _subcomplexe, *a *subunit (ATP6) and *b *subunit (ATP4), were up-regulated under hyperoxia exposure, which is in favour of a mitochondrial respiration. To adapt to hyperoxia, *P. falciparum *seems switch from anaerobic glycolysis to aerobic respiratory metabolism (Figure 7).

In addition to mitochondrial metabolism, the digestive vacuole is another ROS source organelle in *P. falciparum *during haemoglobin digestion [10]. Haemoglobin digestion is optimal at an acidic pH, which is also necessary for protease activity. To provide the acidic environment in the digestive vacuole, haemoglobin digestion stimulates ATP consumption by the V-type H^+^-ATPase pump [66-68]. In microarray analysis, the V-type ATPase complex [PlasmoDB: *MAL13P1.271, PF13_0034, PFE0965c, PF11_0412, PF13_0227, PF13_0065*], a membrane transporter, was found to be down-regulated under hyperoxia. Therefore, *P. falciparum *exposed to hyperoxia could generate a pH change in the digestive vacuole responsible for vacuolar protease activity. Additionally, beta-globin accumulation occurred in hyperoxic conditions. This beta-globin accumulation could result from a decline in protease activity. To test these two hypotheses, transcripts of the proteases involved in beta-globin degradation were quantified. Reduced expression of four genes involved in haemoglobin degradation into AAs (*plasmepsin 2 *[PlasmoDB: *PF14_0077*], *falcipain 2 putative *[PlasmoDB: *PF11_0161*], *falcipain 2 precursor *[PlasmoDB: *PF11_0165*] and *falcipain 3 *[PlasmoDB: *PF11_0162*]) was validated using qRT-PCR. In 2002, Oliveira *et al *hypothesized that blood-feeding parasites reduced their mitochondrial function to compensate for ROS generation from the digestive vacuole [10].

Moreover, catalase [GenBank: gi|4557014], a human protein, was found concentrated in the digestive vacuole [69]. As the *P. falciparum *genome does not contain a catalase gene, the parasite may import human catalase to detoxify H_2_O_2 _generated by oxidation of haem under stress conditions. Collectively, metabolism in the *P. falciparum *digestive vacuole would be perturbed in response to hyperoxia, and ROS production would be slowed (Figure 7).

## Conclusions

Two complementary analytic approaches were used to investigate the response of *P. falciparum *to hyperoxia; (i) a transcriptomic study allowed us to detect whole parasite transcripts, and (ii) a proteomic study identified proteins significantly altered via post-translational modifications and accumulated host proteins. Based on all these results and according to published data mining [29,46], a schematic representation of the adaptive response of *P. falciparum *following hyperoxia exposure was proposed (Figure 7). In order to prove this representation, further biochemical approaches would be required.

Hyperoxia exposure induces metabolic adaptations in *P. falciparum*. These adaptations seem to involve, at least, two parasite organelles, the digestive vacuole and the mitochondrion, both sources of ROS production. To preserve parasite integrity from oxidative stress, all these data suggest that the glycolysis pathway is suppressed in favour of respiratory metabolism and that digestive vacuole metabolism is slowed. Campanale *et al *demonstrated that stress caused by haemoglobin digestion modulates the glycolytic pathway [70]. Highly active mitochondria release H_2_O_2_, which interacts with pro-oxidant products (free iron and haem) in the digestive vacuole. These two ROS sources could be potentially synergistic. The equilibrium of oxidative stress is vital for the parasite; indeed, Hsps could be regulated to facilitate adaptation of parasite to environmental stress as observed in many organisms [56].

The knowledge of the metabolic pathways involved in stress responses to environmental conditions is fundamental to understanding the mechanisms of parasite adaptation. This study provides a starting point for investigations into new anti-malarial treatments, particularly drugs associated with hyperbaric oxygen therapy [34], which has been successfully used to treat other infections [71].

## List of abbreviations

2D-DIGE: 2D-differential-gel-electrophoresis; 2-DE: 2-Dimensional electrophoresis; AA: amino acid; ACN: acetonitrile; BVA: biological variance analysis; Ct: cycle threshold; CQ: chloroquine; Cy-2: cyanine 2; Cy-3: cyanine 3; Cy-5: cyanine 5; DHA: dihydroartemisinin; DHO: dihydroorotate; DHOD: dihydroorotate dehydrogenase; DIA: differential in-gel analysis; FC: fold change; FIX: ferriprotoporphyrin IX; *gapdh*: glyceraldehyde-3-phosphate dehydrogenase; GSEA: Gene Set Enrichment Analysis; *gst*: glutathione S-transferase; H_2_O_2_: hydrogen peroxide; HBO: hyperbaric oxygen therapy; *hsp*: heat shock protein; IEF: isoelectric focusing; MS: mass spectrometry; Nano LC-MS/MS: nanoscale capillary liquid chromatography-tandem mass spectrometry; O^-^_2_: superoxide anions; OH^-^: hydroxyl radicals; O_3_: ozone; *P. falciparum: Plasmodium falciparum*; PMT: photomultiplier tube; NPPs: new permeation pathways; qRT-PCR: quantitative real time PCR; Q-TOF: quadrupole orthogonal acceleration time-of-flight; RBC: red blood cells; ROS: reactive oxygen species, *rpa1*: replication protein A1.

## Competing interests

The authors declare that they have no competing interests.

## Authors' contributions

MTM conceived the study and the design, carried out microarray and proteomic studies, participated in bioinformatics analyses and wrote the manuscript. LA carried out proteomic studies, conducted proteomic statistical analyses and revised the manuscript. JD helped in the design of molecular studies and revised the manuscript. YL participated in the microarray design and conducted statistical and bioinformatics analyses. MB, MP and PF carried out mass spectrometry identifications. YJ conceived the study and edited the manuscript. DP initiated the project, designed the method, participated in the analyses and revised the manuscript. All authors read and approved the final manuscript.

## Supplementary Material

Additional file 1 **Primers sequence using real-time qRT-PCR**. Click here for file

Additional file 2 **Single-Peptide-Based Protein Identifications**. Click here for file

Additional file 3 **Raw data microarray at 8 hours time point under hyperoxia versus normoxia conditions on synchronized parasites**. Click here for file

Additional file 4 **List of altered genes following hyperoxia treatment using GSEA data and PS 6.0 software**. Click here for file

Additional file 5 **MS/MS peptide sequences, respective gi number, gene ID and master spot number of proteins identified from the differential 2-D DIGE analysis following hyperoxia exposure of P. falciparum**. Click here for file

Additional file 6 **Raw data microarray at 4 hours time point under hyperoxia versus normoxia conditions on synchronized parasites**. Click here for file
