# Supplementary material for: Global response of Plasmodium falciparum to hyperoxia: a combined transcriptomic and proteomic approach
Source: Malar J. 2011 Jan 11;10:4. doi: 10.1186/1475-2875-10-4 (PMC3030542; doi:10.1186/1475-2875-10-4)
Supplement: Additional file 1 — Primers sequence using real-time qRT-PCR. [file 1475-2875-10-4-S1.DOC]

**Additional** **file 1:** Primers sequence using real-time qRT-PCR.

| **Accession Number** ^a^ | **Name Primers** | **Forward (F) and reverse (R) sequencing primers** |
| --- | --- | --- |
| MAL7_18Sa | *rRNA18S*/F  *rRNA18S*/R | 5’ GCTGACTACGTCCCTGCCC 3’  5’ ACAATTCATCATATCTTTCAATCGGTA 3’ |
| PF14_0077 | *plasmepsin 2*/F | 5’ TTAGATGTTATCAAAGTCCCATTCTTACC 3’ |
|  | *plasmepsin 2*/R | 5’ CAGGTTCTAATGTGTATTTACCATTTTCTG 3’ |
| PF11_0161 | *falcipain 2p61*/F | 5’ GCAGCTTATGATTGGAGATTACATAGTG 3’ |
|  | *falcipain 2p61*/R | 5’ AGCATATTGTGATTCTACGGAACCTATACT 3’ |
| PF11_0165 | *falcipain 2p65*/F | 5’ AGAAGGTATTTTCGATGGAGAATGTG 3’ |
|  | *falcipain 2p65*/R | 5’ TGTTGTCCCCATGAGTTCTTAATTATATAA 3’ |
| PF11_0162 | *falcipain 3*/F | 5’ GAGAATGTGGAGCAGCACCAA 3’ |
|  | *falcipain 3*/R | 5’ CAGATCCCCATGGTTTTTAATGATATAA 3’ |
| PF14_0598 | *gapdh*/F | 5’ GAAGGTCCACTTAAAGGAATCTTAGGAT 3’ |
|  | *gapdh*/R | 5’ TTCATGTCAAAGATTGATGATCTGTTATC 3’ |
| PF10_0245 | *gf6p*/F | 5’ AGAACTGGCTTATATACATTGTGAAGGTTT 3’ |
|  | *gf6p* /R | 5’ TTACAGGGATATTGTCTTCACCACCTA 3’ |
| PF14_0187 | *gst*/F | 5’ AACGGTGATGCTTTTGTTGAATT 3’ |
|  | *gst* /R | 5’ GCTTTGAGCTAATATCAAATCTCCAA 3’ |
| PF11_0087 | *rad51*/F | 5’ TAACCAAGTCGTTGCCAAGGT 3’ |
|  | *rad51*/R | 5’ CATGAGCTATTATGTTTCCACCTATAGG 3’ |
| PFL1110c | *pkareg*/F | 5’ GGAGAATTAGCTCTTCTCAAAAATAAACC 3’ |
|  | *pkareg* /R | 5’ GTCCTAATAATCTTTTGAAACTTTTTCTATCTAAATA 3’ |
| PFI1685w | *pkacat*/F | 5’ AGAAATTTTATTGAACGTCGGACAT 3’ |
|  | *pkacat* /R | 5’ AGGGTTCATTCGCATAAAAAGG 3’ |
| PF10_0059 | *atp6/F* | 5’ AAGCAAAACAATATTCTTCTTATCGATT 3’ |
|  | *atp6/R* | 5’ TGTGTAAAGATTTTGATTTGTCATAGGAA 3’ |
| MAL7P1.13 | *atp4/F* | 5’ ATTTACAGTAAACAATTTTCTCTCAGTCAAA 3’ |
|  | *atp4/R* | 5’ ATACTGAAATGTAAGCCACTTAATCTTTTC 3’ |

^a^ Accession Number from Plasmodium Genome Resource database [www.plasmodb.org]

Foodnotes: *gapdh*: glyceraldehyde-3-phosphate dehydrogenase - *gf6p*: glutamine-fructose-6-phosphate transaminase - *gst*: glutathione S-transferase - *pka*: Protein kinase A - *atp6*: mitochondrial ATP synthase F0 *a* subunit – *atp4*: mitochondrial ATP synthase F0 *b* subunit.
