# Supplementary material for: Global response of Plasmodium falciparum to hyperoxia: a combined transcriptomic and proteomic approach
Source: Malar J. 2011 Jan 11;10:4. doi: 10.1186/1475-2875-10-4 (PMC3030542; doi:10.1186/1475-2875-10-4)
Supplement: Additional file 2 — Single-Peptide-Based Protein Identifications. [file 1475-2875-10-4-S2.PPT]

## Slide 1
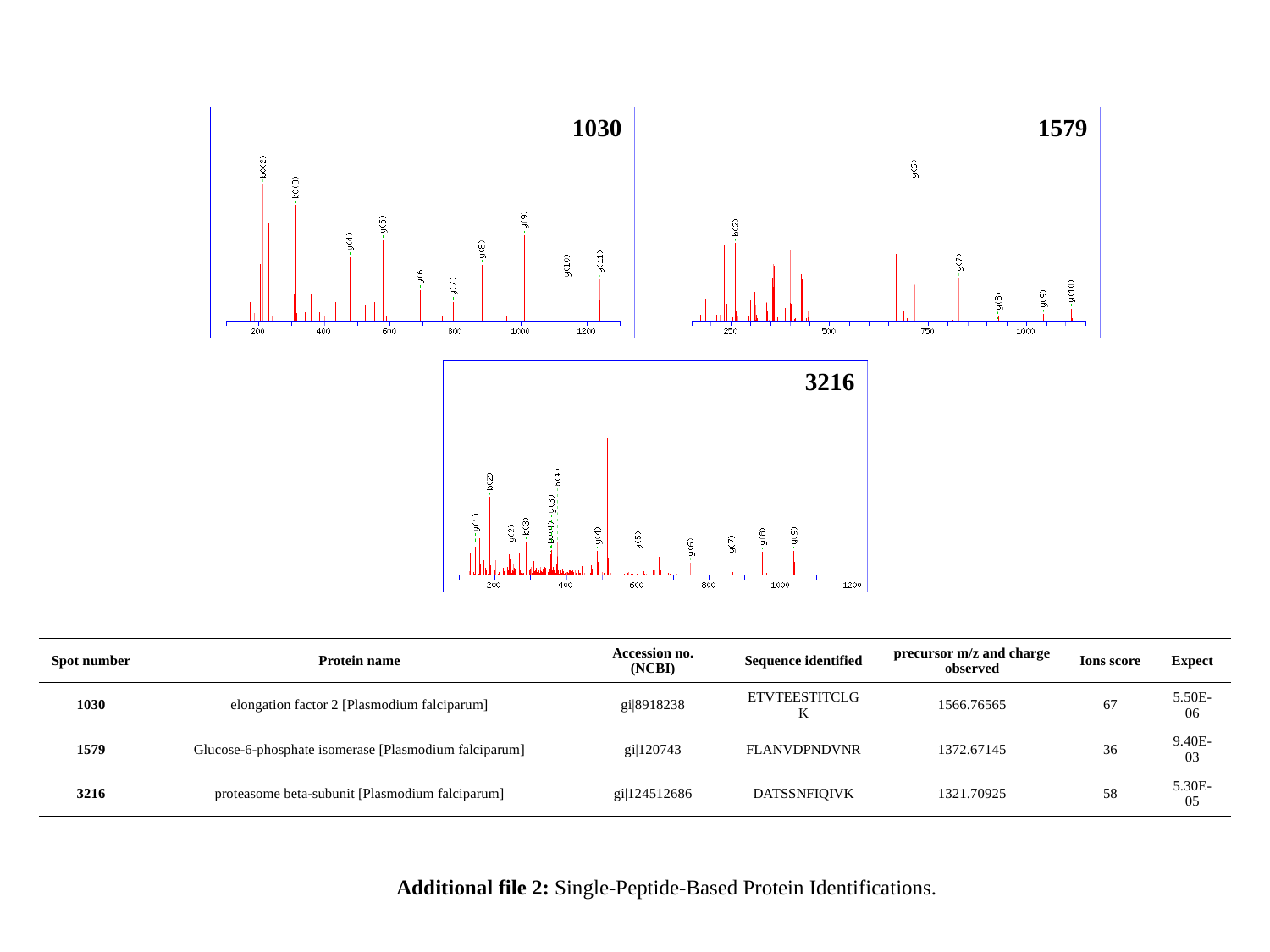

1030
1579
3216
| Spot number | Protein name | Accession no. (NCBI) | Sequence identified | precursor m/z and charge observed | Ions score | Expect |
| --- | --- | --- | --- | --- | --- | --- |
| 1030 | elongation factor 2 [Plasmodium falciparum] | gi|8918238 | ETVTEESTITCLGK | 1566.76565 | 67 | 5.50E-06 |
| 1579 | Glucose-6-phosphate isomerase [Plasmodium falciparum] | gi|120743 | FLANVDPNDVNR | 1372.67145 | 36 | 9.40E-03 |
| 3216 | proteasome beta-subunit [Plasmodium falciparum] | gi|124512686 | DATSSNFIQIVK | 1321.70925 | 58 | 5.30E-05 |
Additional file 2: Single-Peptide-Based Protein Identifications.
