# Supplementary material for: Global response of Plasmodium falciparum to hyperoxia: a combined transcriptomic and proteomic approach
Source: Malar J. 2011 Jan 11;10:4. doi: 10.1186/1475-2875-10-4 (PMC3030542; doi:10.1186/1475-2875-10-4)
Supplement: Additional file 5 — MS/MS peptide sequences, respective gi number, gene ID and master spot number of proteins identified from the differential 2-D DIGE analysis following hyperoxia exposure of P. falciparum. [file 1475-2875-10-4-S5.DOC]

**Additional file 5:** MS/MS peptide sequences, respective gi number, gene ID and master spot number of proteins identified from the differential 2-D DIGE analysis following hyperoxia exposure of *P. falciparum*.

| **gi number** | **Gene ID** | Master spot number | **MS/MS**  **Peptide Sequences** |
| --- | --- | --- | --- |
| **P. falciparum** | | | |
| Translation | | | |
| gi|124512420 | MAL8P1.69 | 3289 | R.CDCTYR.S  K.LAEQAER.Y  R.YDEMADAMR.T  R.TLVEQCVNNDKDELTVEER.N  R.IISSVEQK.E  K.NVAATYR.K  K.LIPNTSESESK.V  R.AFDDAITEFDNVSEDSYK.D  K.DSTLIMQLLR.D |
| gi|124513850 | PF13_0304 | 2047 | R.TLIEALDTMEPPK.R  K.IGGIGTVPVGR.V  R.GYVASDTK.N  K.FLNIDSK.I  K.VVEENPK.A  R.QTIAVGIIK.S  K.KEPGAVTAK.A |
| gi|8918238 | PF14_0486 | 1030 | R.ETVTEESTITCLGK.S |
| gi|124810293 | PF14_0655 | 2268 | R.ELAQQIQK.V  K.LFILDEADEMLSR.G  K.KDELTLEGIR.Q  K.DELTLEGIR.Q  R.VLVTTDLLAR.G |
| Parasitophorous vacuolar membrane Transporter | | | |
| gi|124810348 | PF14_0678 | 3061 | K.DPISLTIK.D  R.EIVGDNTIEK.K  R.EIVGDNTIEKK.T |
|  |  | 3062 | R.EIVGDNTIEK.K  R.EIVGDNTIEKK.T  K.LRQDPSLIVAK.I  R.QDPSLIVAK.I |
| Glycolysis | | | |
| gi|124809201 | PF14_0341 | 1579 | R.FLANVDPNDVNR.A |
| gi|124810131 | PF14_0598 | 2867 | K.LGINGFGR.I  K.DPSQIPWGK.C  K.ELASSHLK.G  K.KVIMSAPPK.D  K.VIMSAPPK.D  K.QLIVSNASCTTN.C  K.QLIVSNASCTTNCLAPLAK.V  R.CALSNIIPASTGAAK.A  K.VLPELNGK.L  R.VPIGTVSVVDLVCR.L  K.YEEVALEIK.K  K.KAAEGPLK.G  K.AGLALNDNFFK.L |
| Chaperone-assisted protein folding | | | |
| gi|124512406 | PF08_0054 | 1314 | R.TTPSYVAFTDTER.L  R.NPENTVFDAK.R  K.FTESSVQSDMK.H  K.NAVITVPAYFNDSQR.Q  K.DAGTIAGLNVMR.I  R.LVNFCVEDFK.R  R.FEELCIDYFR.D  R.NTTIPAKK.S  R.LSQDEIDR.M  K.LQPAEIETCMK.T  K.EAESVCAPIMSK.I |
|  |  | 1326 | R.TTPSYVAFTDTER.L  R.NPENTVFDAK.R  K.FTESSVQSDMK.H  K.ATAGDTHLGGEDFDNR.L  R.LVNFCVEDFK.R  R.NTTIPAKK.S  R.LSQDEIDR.M  K.LQPAEIETCMK.T  K.EAESVCAPIMSK.I |
| gi|505340 | PF07_0029 | 1028 | M.STETFAFNADIR.Q  R.ELISNASDALDK.I  K.LSAEPEFFIR.I  K.TNNTLTIEDSGIGMTK.N  K.NDLINNLGTIAR.S  K.VEDVTEELENAEK.K  K.ALLFIPK.R  K.GVVDSEDLPLNISR.E  K.SGDEMIGLK.E |
| Amino acids metabolism | | | |
| gi|86170756 | PFF0435w | 2217 | R.TLGCVSASTDK.K  K.LCENADK.L  K.LGAPFLQNLK.E  K.GLLCAIEFK.N  K.KEPGAVTAK.A |
| gi|124513590 | MAL13P1.214 | 3347 | M.TLIENLNSDK.T  K.ILSDIELNENSK.V  N.IVNMANER.V  K.IIFEANDILTK.E  K.EFPENNFDLIYSR.D  K.FISLDDGWSR.K |
|  |  | 3372 | M.TLIENLNSDK.T  K.ILSDIELNENSK.V  K.IIFEANDILTK.E  K.NVVSK.D |
|  |  | 3379 | M.TLIENLNSDK.T  K.ILSDIELNENSK.V  K.IIFEANDILTK.E |
|  |  | 3503 | K.ILSDIELNENSK.V  K.IIFEANDILTK.E |
|  |  | 3385 | M.TLIENLNSDK.T  K.TFLENNQYTDEGVK.V  K.ILSDIELNENSK.V  N.IVNMANER.V  K.IIFEANDILTK.E  K.EFPENNFDLIYSR.D  K.ENWDDEFK.E  K.FISLDDGWSR.K |
| Proteasome-mediated proteolysis | | | |
| gi|124512686 | MAL8P1.142 | 3216 | R.DATSSNFIQIVK.V |
| gi|124513790 | MAL13P1.270 | 3358 | K.SSNFAVLAVEK.K  K.IVQEFLEK.N  K.AIFEVVELSSK.N  K.NVEVALLTEK.D |
| ***Homo sapiens*** | | | |
| Oxygen transporter | | | |
| gi|183817 |  | 3451 | K.VNVDEVGGEALGR.L  R.FFESFGDLSTPDAVMGNPK V |
|  |  | 3576 | K.VNVDEVGGEALGR.L  R.FFESFGDLSTPDAVMGNPK V |
| Antioxidant metabolism | | | |
| gi|4502517 |  | 3431 | K.NGPEQWSK.L  K.YSSLAEAASK.A  K.ADGLAVIGVLMK.V  K.VLDALQAIK.T |
|  |  | 3444 | K.YSSLAEAASK.A  K.ADGLAVIGVLMK.V  K.VLDALQAIK.T |
|  |  | 3455 | K.NGPEQWSK.L  K.LYPIANGNNQSPVDIK.T  K.GGPFSDSYR.L  K.YSSLAEAASK.A  K.ADGLAVIGVLMK.V  K.VLDALQAIK.T |
| gi|4557014 |  | 1648 | K.ADVLTTGAGNPVGDK.L  K.LNVITVGPR.G  K.NLSVEDAAR.L  R.DLFNAIATGK.Y  K.DYPLIPVGK.L |
| gi|16306550 |  | 2451 | K.SQPEPLVVK.G  K.LVLPSLISSR.I  R.IYVVDVGSEPR.A |
| gi|168985379 |  | 2156 | K.EMLAAACQMFLGK.T  R.AQQVAVQEQEIAR.R  K.ITLVSSGSGTMGAAK.V |
| Glycolysis | | | |
| gi|31645 |  | 2804 | K.LVINGNPITIFQER.D  R.GALQNIIPASTGAAK.A  K.LTGMAFR.V  R.VPTANVSVVDLTCR.L  K.QASEGPLK.G.L |
|  |  | 2806 | K.LVINGNPITIFQER.D  R.GALQNIIPASTGAAK.A  K.LTGMAFR.V  R.VPTANVSVVDLTCR.L  K.QASEGPLK.G.L |
|  |  | 2807 | R.GALQNIIPASTGAAK.A  R.VPTANVSVVDLTCR.L  K.QASEGPLK.G.L |
|  |  | 2853 | K.LVINGNPITIFQER.D  K.LTGMAFR.V  K.QASEGPLK.G.L |
|  |  | 2854 | K.LVINGNPITIFQER.D  R.GALQNIIPASTGAAK.A  K.LTGMAFR.V  K.QASEGPLK.G.L |
|  |  | 2856 | R.GALQNIIPASTGAAK.A  R.VPTANVSVVDLTCR.L |
